# Treatment Principle Based on the Clinical Staging of Pharyngocutaneous Fistula

**DOI:** 10.1155/2020/2373549

**Published:** 2020-05-14

**Authors:** Wei Sun, Ren-Qiang Ma, Wei-Ping Wen, Xiao-Lin Zhu

**Affiliations:** ^1^Department of Otorhinolaryngology Head and Neck Surgery, The First Affiliated Hospital of Sun Yat-sen University, Guangzhou 510080, China; ^2^Institute of Otorhinolaryngology Head and Neck Surgery, Sun Yat-sen University, Guangzhou 510080, China

## Abstract

**Objective:**

Studies on factors affecting pharyngocutaneous fistulas (PCFs) and PCF repair methods have been widely reported. However, the healing phases of PCF are unclear, and their elucidation could guide clinical treatment.

**Methods:**

Clinical stages of the PCF healing process were identified by a retrospective study of 39 patients with head and neck cancer who developed a PCF.

**Results:**

Different conservative treatments were performed in turn according to three defined stages of the PCF healing process: stage I (drainage and debriding period), stage II (pressure dressing period), and stage III (healing period). A 7-day course of antibiotic therapy was only performed in stage I in 23 patients. The PCF was cured in 30 (76.9%) of 39 patients; the remaining 9 patients underwent subsequent surgical interventions for PCF healing.

**Conclusion:**

The three stages of PCF healing have a certain reference value in guiding clinical treatments. Moreover, antibiotics should be used in stage I when signs of infection are present, but they should not be used in all three phases of conservative treatment.

## 1. Introduction

The occurrence of a pharyngocutaneous fistula (PCF) after head and neck cancer surgery is a serious complication. PCF is defined as leakage of saliva through the pharyngeal closure site to the skin. The incidence of PCF varies from 9% to 25% in the last decade [1]. PCF affects the ability to perform timely postoperative radiotherapy, prolongs hospitalization time, and increases the economic burden.

Preoperative treatment and intraoperative repair of the pharyngeal cavity are important to prevent the occurrence of PCF [2–4]. Although the incidence of PCF has been reduced with the use of adjacent flaps, pectoralis major musculocutaneous flaps, or free flaps to repair the pharynx, PCF still occurs in some patients [2–5]. Studies of factors affecting PCF and various repair methods for curing PCF have been widely reported [1–13]. However, no study has addressed the different clinical phases of PCF healing, which may provide guidance for the clinical treatment of PCF.

In the present study, three clinical stages of the PCF healing process were identified according to the characteristics of 39 patients with head and neck cancer who developed a PCF after total laryngectomy or total pharyngolaryngectomy. Moreover, satisfactory outcomes were achieved after performing sequential conservative treatments on the basis of each PCF stage.

## 2. Materials and Methods

### 2.1. Patients

The main clinical and pathologic characteristics of the patients are presented in Table 1. Clinical staging of the tumors was performed according to the 7th edition of the Union for International Cancer Control (UICC 2010) tumor-node-metastasis classification of malignant tumors [14]. All clinicopathological data were obtained from the hospital's database with the approval of the Ethics Committee of The First Affiliated Hospital of Sun Yat-sen University, Guangzhou, China.

Total laryngectomy was performed in 260 patients with laryngeal squamous cell carcinoma (SCC) and total pharyngolaryngectomy was performed in 91 patients with hypopharyngeal SCC in the First Affiliated Hospital of Sun Yat-Sen University from January 1998 to May 2018. After the operation, PCF was found in 18 patients with laryngeal SCC and 21 patients with hypopharyngeal SCC. The PCF in 39 patients was treated conservatively.

### 2.2. Treatments Based on Clinical Staging of PCF

Three different clinical stages of the PCF healing process were identified.

#### 2.2.1. Drainage and Debriding Period (Stage I)

This period usually lasted for 1 week after the appearance of the PCF. Drainage and debriding of necrotic tissues was mainly carried out during this period. Secretions from the PCF were sent for bacterial culture and drug sensitivity testing if signs of infection were observed (e.g., redness and swelling around the PCF). Broad-spectrum antibiotics were administered to patients before obtaining the results of the drug sensitivity tests, and the broad-spectrum antibiotics were then changed to sensitive antibiotics if necessary according to the results of the drug sensitivity tests. No antibiotics were administered to patients without signs of infection. The number of dressing changes varied from one to four times daily depending on the amount of secretions from the PCF and the degree to which the dressing stayed dry. In addition, supportive treatments were applied according to the nutritional status of the patients; e.g., albumin was given to patients with albuminemia (<30 g/L), blood transfusion was performed when the hemoglobin level was <80 g/L, and a variety of amino acids and fat emulsion could be given when a negative nitrogen balance occurred.

#### 2.2.2. Pressure Dressing Period (Stage II)

This period usually lasted for 1 to 2 weeks after completion of stage I. During this period, pressure dressings were applied. The redness and swelling around the PCF disappeared, and the secretions had no odor. To reduce the saliva entering the PCF cavity, which could affect the development of granulation tissue, the location of the internal opening of the PCF was wrapped with gauze outside the neck, and Vaseline or iodoform gauze was inserted into the PCF cavity to prevent stenosis of the external opening of the PCF. The pale granulation tissue that formed in the PCF cavity could be scratched and removed, and peptides or insulin could be used to promote the healing of the PCF. The number of dressing changes was minimized to once every 2 to 4 days according to the degree to which the dressing stayed dry.

#### 2.2.3. Healing Period (Stage III)

Most of the PCFs healed within 1 to 3 weeks in this period. Surgical intervention was needed if the PCF had not healed after more than 6 weeks of conservative treatment. In the present study, nine patients with a PCF underwent surgical treatments involving simple fistula resection in one, local cervical flap repair in two, and pectoralis major myocutaneous flap repair in four (two patients underwent two operations: local cervical flap repair and pectoralis major myocutaneous flap repair).

### 2.3. Statistical Analysis

All data were stored in a database and then analyzed using SPSS for Windows, version 13 (SPSS Inc., Chicago, IL, USA). The chi-square test (crosstabs) was used to compare the incidence of PCF, and the body temperature was analyzed using an independent-samples *t*-test. A *p* value of <0.05 was considered statistically significant.

## 3. Results

The bacterial culture results showed that bacteria were absent in the PCF secretions of 7 patients and present in the PCF secretions of 24 patients. The species of bacteria are shown in Table 2. The PCF secretions from eight patients without signs of infection were not sent for bacterial culture.

The overall incidence of postoperative PCF was 11.1% (39/351). The incidence of PCF (23.0%) after surgical treatment of hypopharyngeal SCC was significantly higher than that after surgical treatment of laryngeal SCC (6.9%) (*p* < 0.01). In the cohort of patients with laryngeal SCC and hypopharyngeal SCC, the incidence of postoperative PCF was significantly different between patients with and without preoperative chemoradiotherapy (*p* < 0.05). However, among patients with laryngeal SCC, the incidence of postoperative PCF was not significantly different between patients with and without chemoradiotherapy (*p* > 0.05). Only among patients with hypopharyngeal SCC, the incidence of postoperative PCF was significantly different between patients with and without chemoradiotherapy (*p* < 0.05) (Table 3).

PCFs occurred 4 to 30 days (median, 9 days) after laryngectomy or pharyngolaryngectomy. There was no significant difference in the time of PCF occurrence between patients with laryngeal SCC (range, 4–30 days; median, 10 days) and those with hypopharyngeal SCC (range, 5–20 days; median, 8 days) (*p* > 0.05). The body temperature was higher before than after the occurrence of PCF (37.2°C ± 0.6°C vs. 36.9°C ± 0.5°C, respectively; *p* < 0.05). The cure rate and time until cure of conservatively treated PCFs were 76.9% (30/39) and 16 to 60 days (median, 26.5 days), respectively. The time until cure was not significantly different between patients with laryngeal SCC and hypopharyngeal SCC (*p* > 0.05).

According to the diameter of the internal opening of the PCF, the PCFs were categorized as large (internal opening diameter of >2 cm) in 12 patients, moderate (internal opening diameter of 1–2 cm) in 16 patients, and small (internal opening diameter of <1 cm) in 6 patients, and the internal opening diameter was unknown in 5 patients. Moreover, each PCF was categorized as either a single PCF with one internal opening (29 patients) or a complex PCF with two or more internal openings (10 patients). Eight patients with a large PCF were cured by conservative treatment, and four were cured by surgical treatment. Thirteen patients with a moderate PCF were cured by conservative treatment, and three were cured by surgical treatment. Four patients with a small PCF and five patients with an unknown internal opening diameter were cured by conservative treatment. Two patients with a small PCF were cured by surgical treatment.

## 4. Discussion

The occurrence of PCF is associated with a variety of factors [1–12], including preoperative chemoradiotherapy, nutritional status, and intraoperative pharyngeal suture technique. However, the relationship between the occurrence of PCF and infection remains controversial [5, 6]. The present study suggests no obvious relationship between infection and the occurrence of PCF. This conclusion can be attributed to the following clinical observations. (1) The spectrum of pathogenic bacteria isolated from the internal secretions from the PCF was basically the same as the bacterial spectrum of the human nasal cavity [15–17]. (2) Although the difference in body temperature before and after PCF was statistically significant, the maximum body temperature before and after PCF was 37.2°C and 36.9°C, respectively, which did not reach the criteria for clinical infection. (3) The peculiar odor of the internal secretions from the PCF was caused by protein necrosis and differed from the odor of purulent discharge. (4) Most of the PCFs occurred about 10 days postoperatively (median, 8 days), which coincided with the time of suture-associated necrosis at the mucosal margin of the wound and white scar dropping (similar to the process of wound healing after tonsillectomy). (5) The results of the present study support the reasonableness of administering antibiotics only to patients with signs of infection in PCF stage I. All of these clinical observations suggest that antibiotic treatment is not necessary for every case of PCF and is not necessary during the whole course of the PCF.

Few studies to date have focused on conservative treatment based on the PCF healing phases. In the present study, we proposed that the healing process of PCF could be divided into three stages according to the time of occurrence, wound condition, and conservative treatment measures. Different conservative treatment measures were performed in each stage. In detail, within 1 week after the occurrence of PCF (defined as stage I), debriding and drainage of necrotic tissues and secretions were mainly performed. Antibiotics were administered according to the drug sensitivity test of the PCF secretions, and supportive treatment was carried out according to the patient's systemic nutritional status. Within 1 to 2 weeks after stage I (defined as stage II), the key point of treatment was the prevention of salivary flow into the PCF via pressure dressing. Continuous treatment after stage II usually lasted for 1 to 3 weeks, and most of the PCFs could be cured during this period (defined as stage III). In the present study, the PCF healing time ranged from 4 to 6 weeks, which is consistent with previous studies [6].

Surgical interventions could be performed if the PCF was not cured. The indications for surgical interventions were as follows: the PCF was not cured after 4 to 6 weeks of conservative treatment, the general state of the patients allowed them to tolerate the surgery, and the important blood vessels were exposed. Different methods of surgical repair could be adopted according to the size of the fistula, including adjacent skin flaps, pectoralis major myocutaneous flaps, and free flaps [6–13].

Negative pressure wound therapy (NPWT) has recently been considered an effective method of the conservative treatment of PCF [18]. NPWT can reduce fistula secretions and interstitial edema as well as promote the growth of granulation tissues by turning the open wound into a closed wound. However, not all PCFs can be treated by NPWT. For example, NPWT is not suitable for fistulas located close to a tracheostoma mainly because it is difficult to maintain an airtight seal. Just as important, NPWT is contraindicated in the following situations: uncontrolled wound infection, necrotic tissues requiring debridement, absence of an interface between the negative-pressure system and the gastrointestinal tract, and presence of a pressure ulcer. Hence, mastering the clinical staging of PCF as described in the present study is also important for conservative treatment of PCFs when NPWT cannot be performed.

In summary, according to the three different stages of the healing process of PCFs (defined as the drainage and debriding period (stage I), pressure dressing period (stage II), and healing period (stage III)), conservative treatments were carried out in turn with satisfactory outcomes. The present study suggests that this staging system of PCFs can be adopted in the clinical setting. Moreover, antibiotics should be used in the early stage (stage I) of PCF when signs of infection are present, but antibiotics are not needed throughout all three phases of conservative treatment.

## Figures and Tables

**Table 1:** Main clinical and pathologic characteristics of laryngeal SCC and hypopharyngeal SCC.

Cancer	Cases	III	IVA		IVB
		*T*_3_N_0-1_*M*_0_	T4_a_N_0-1_*M*_0_	T_4a_N_2_*M*_0_	T_4a_N_3_*M*_0_
Laryngeal SCC	18	8	9	1	0
Hypopharyngeal SCC	21	8	7	5	1

**Table 2:** Bacterial culture results from secretions of 24 PCFs.

Names of bacteria	Cases
*Escherichia coli*	7
*Pseudomonas aeruginosa*	5
*Staphylococcus epidermidis*	5
Pathogenic Streptococcus	3
*Branhamella catarrhalis*	1
*Candida tropicalis*	1
*Bacillus proteus* and *Pseudomonas aeruginosa*	1
*Pseudomonas aeruginosa* and *Escherichia coli*	1

**Table 3:** Incidence of postoperative PCF in patients with laryngeal and hypopharyngeal SCC with or without preoperative chemoradiotherapy.

PCF	Malignant tumor		*P*	Laryngeal SCC		*P*	Hypopharyngeal SCC		*P*
	Yes	No		Yes	No		Yes	No	
Yes	9	30	0.038	2	16	1.000	7	14	0.047
No	34	278		25	217		9	61	

Chi-square tests (crosstabs).

## Data Availability

The data used to support the findings of this study are available from the corresponding author upon request (e-mail: zhuxlin2@mail.sysu.edu.cn).
